# Pharmacokinetics of Novel Furoxan/Coumarin Hybrids in Rats Using LC-MS/MS Method and Physiologically Based Pharmacokinetic Model

**DOI:** 10.3390/molecules28020837

**Published:** 2023-01-13

**Authors:** Yawen Yuan, Zhihong Li, Ke Wang, Shunguo Zhang, Qingfeng He, Lucy Liu, Zhijia Tang, Xiao Zhu, Ying Chen, Weimin Cai, Chao Peng, Xiaoqiang Xiang

**Affiliations:** 1 Department of Pharmacy, Shanghai Children’s Medical Center, School of Medicine, Shanghai Jiao Tong University, Shanghai 200127, China; 2 Department of Clinical Pharmacy and Pharmacy Administration, School of Pharmacy, Fudan University, Shanghai 201203, China; 3 National Facility for Protein Science in Shanghai, Zhangjiang Lab, Shanghai Advanced Research Institute, Chinese Academy of Sciences, Shanghai 201210, China; 4 Department of Medicinal Chemistry, School of Pharmacy, Fudan University, Shanghai 201203, China

**Keywords:** furoxan/coumarin hybrids, preclinical pharmacokinetics, LC-MS/MS method, physiologically based pharmacokinetic model

## Abstract

Novel furoxan/coumarin hybrids were synthesized, and pharmacologic studies showed that the compounds displayed potent antiproliferation activities via downregulating both the phosphatidylinositide 3-kinase (PI3K) pathway and the mitogen-activated protein kinase (MAPK) pathway. To investigate the preclinical pharmacokinetic (PK) properties of three candidate compounds (CY-14S-4A83, CY-16S-4A43, and CY-16S-4A93), liquid chromatography, in tandem with the mass spectrometry LC-MS/MS method, was developed and validated for the simultaneous determination of these compounds. The absorption, distribution, metabolism, and excretion (ADME) properties were investigated in in vitro studies and in rats. Meanwhile, physiologically based pharmacokinetic (PBPK) models were constructed using only in vitro data to obtain detailed PK information. Good linearity was observed over the concentration range of 0.01–1.0 μg/mL. The free drug fraction (*f*_u_) values of the compounds were less than 3%, and the clearance (CL) values were 414.5 ± 145.7 mL/h/kg, 2624.6 ± 648.4 mL/h/kg, and 500.6 ± 195.2 mL/h/kg, respectively. The predicted peak plasma concentration (C_max_) and the area under the concentration-time curve (AUC) were overestimated for the CY-16S-4A43 PBPK model compared with the experimental ones (fold error > 2), suggesting that tissue accumulation and additional elimination pathways may exist. In conclusion, the LC-MS/MS method was successively applied in the preclinical PK studies, and the detailed information from PBPK modeling may improve decision-making in subsequent new drug development.

## 1. Introduction

PI3K and MAPK pathways are two fundamental intracellular signaling cascades, with multiple downstream proteins involved [1,2]. Some of the proteins, such as mitogen-activated protein kinase-extracellular signal-regulated kinase (MEK), extracellular signal-regulated kinase (ERK), and protein kinase B (as known as AKT), are commonly altered in tumors. Therefore, many targeting pharmaceutical agents based on the proteins or pathway have been developed to treat cancers, including non–small cell lung cancer (NSCLC), ovarian cancer, and colorectal cancer [3,4,5,6]. For example, trametinib was approved by the FDA as the first potent MEK/ERK kinase inhibitor to treat metastatic melanoma and NSCLC in combination with dabrafenib [7,8]. Perifosine, an AKT inhibitor, is currently being evaluated in a clinical trial for its effectiveness when combined with temsirolimus for treating recurrent malignant gliomas [9].

Recent years have seen growing interest in novel furoxan-based coumarin derivatives because these compounds exhibit potent cytotoxicity and anticancer activities [10,11]. Although coumarin has become popular thanks to its significant anticoagulant effects, it can also intervene in the MAPK pathway. For instance, 7,8-Dihydroxy-4-methyl coumarin was found to induce A549 human NSCLC cell apoptosis via the partial inhibition of ERK/MARK signaling [12]. Through the same mechanism, xanthoxyletin, a plant-derived coumarin, inhibits human oral squamous carcinoma cell proliferation [13]. Additionally, the synergistic use of furoxan derivatives can further enhance the anticancer activity from coumarin derivatives [14,15]. Furoxan derivatives release nitric oxide (NO) [16], an endothelium-derived relaxing factor (EDRF) found to downregulate the PI3K pathway [17,18,19], demonstrating a potent therapeutic effect on NSCLC and liver cancer [20,21]. In addition, thanks to the upregulating effect of furoxan derivatives on the MAPK pathway [17,18,19], more and more novel coumarin/furoxan hybrids are being synthesized and investigated for clinical applications as new drugs to further improve antitumor activity [22]. For example, we designed and synthesized a series of novel henylsulfonylfuroxan-merging coumarin analogs and evaluated the antitumor activity against the A549, HeLa, A2780, A2780/CDDP, and HUVEC cell lines. Moreover, the antitumor activity was also demonstrated in female mice [23,24]. The results showed that the compounds displayed potent antineoplastic activities with the IC_50_ (half maximal inhibitory concentration) of 0.5–143 nM and low toxicities.

As part of the drug-screening and evaluation system to assess the risk–benefit ratio, preclinical PK studies can help characterize the in vivo behavior of coumarin/furoxan hybrids. Through in vitro or in silico methods, we can quickly obtain several PK parameters, including ADME properties [25,26]. However, experimentally measuring drug exposure in target tissues remains difficult and presents ethical concerns. Since William Russel and Rex Burch proposed the 3Rs principle, namely replacement, reduction, and refinement for animal welfare in 1959, more and more studies have focused on the improvement of animal experimental protocols, which has also brought challenges to traditional PK studies [27,28]. In recent decades, computational technologies have rapidly developed and been increasingly applied in preclinical phases [29,30,31]. As a mathematical model, the PBPK model combines the drug properties and physiological characteristics of the organism [32,33,34,35,36,37], which can simulate the PK process of drugs in vivo. The PBPK model possesses the ability to estimate target tissue concentration, which is highly correlated with drug action. This significant advantage enables PBPK modeling to be used throughout the drug development process [38,39,40]. Moreover, with the rapid development of software and other computer techniques, a well-validated and reliable PBPK model guarantees a relatively accurate prediction of PK properties.

The present work aims to investigate the preclinical PK properties of novel coumarin/furoxan hybrid candidates in the drug-screening process, to provide decision-making information for subsequent new drug development. A quantification analysis method for the simultaneous determination of three candidate compounds (CY-14S-4A83, CY-16S-4A43, and CY-16S-4A93) by LC-MS/MS was developed and validated. The physicochemical and PK properties of the compounds were obtained via experimental and in silico methods. PBPK models were further constructed by using in vitro data alone for determining detailed PK properties, using Simcyp (Sheffield, UK). This preclinical PK study of novel coumarin/furoxan hybrid candidates may provide an example of applying 3Rs in a PK study.

## 2. Results

### 2.1. LC-MS/MS Method Validation

**Figure 1:**
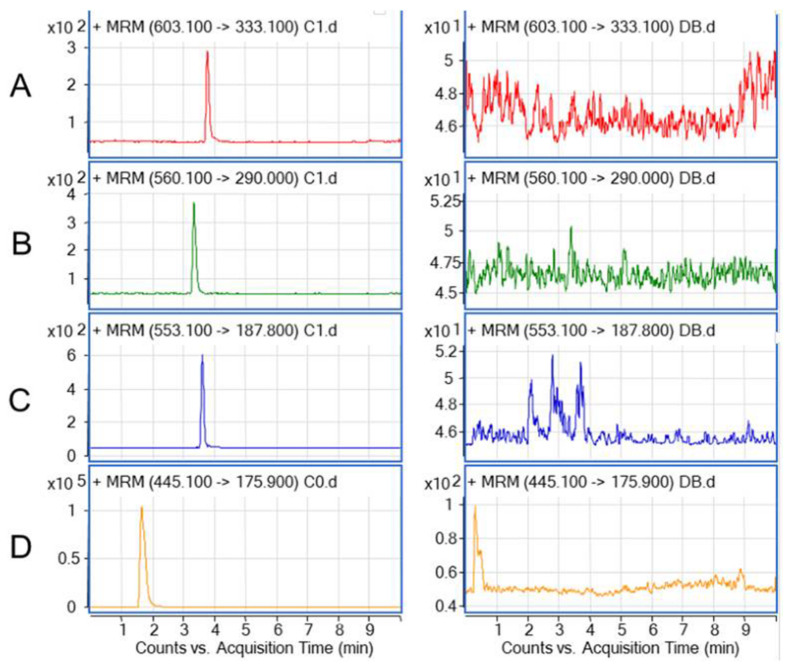
Representative MRM transitions for the chromatograms of (**A**) CY-14S-4A93, (**B**) CY-16S-4A43, (**C**) CY-14S-4A83, and (**D**) IS in rat plasma. Left panel: A, B, and C are LLOQ samples (10 ng/mL) of CY-14S-4A93, CY-16S-4A43, and CY-14S-4A83, respectively and D is a spiked sample (200 ng/mL) of IS. Right panel: blank plasma samples.

Because the compounds all belong to coumarin/furoxan hybrid candidates and their structures are very close, the drug concentration in rats needed to be determined to analyze the PK properties in vivo. That is, the LLOQ of the analysis method should cover a concentration range of 3–5 half-life periods of the compounds in vivo, so the LC-MS/MS method with high sensitivity and specificity was finally selected for quantification. The representative chromatograms of blank rat plasma sample, LLOQ samples, and IS are shown in Figure 1. No significant interfering peaks from the endogenous species were observed at the retention time of the analytes and IS. The results indicated that the UPLC-MS/MS method exhibited good specificity for analytes in rat plasma. The calibration curve was linear over the concentration range of 10–1000 ng/mL, as shown in Table 1, and the R^2^ was greater than 0.99 for all curves, indicating good linear correlation.

The results of intra- and interday precision and accuracy, recovery, matrix effect, stability, and dilution integrity are tabulated in Table 2. For intra- and interday precisions and accuracy, the CV% values calculated for all tested levels (*n* = 6) did not exceed 12.5%, and the RE ranged from 90% to 114.6%, indicating the adequate reliability and reproducibility of the LC-MS/MS method within the analytical range. The extraction recovery of CY-14S-4A83, CY-16S-4A43, and CY-16S-4A93 ranged from 85.16% to 114.37%, and the CV% values were no more than 9%, suggesting acceptable loss in the extraction process of the LC-MS/MS method. The respective matrix factors of CY-14S-4A83, CY-16S-4A43, and CY-16S-4A93 ranged from 86.25% to 113.89%, and the CV% values were less than 13%, indicating that the matrix effect did not significantly affect the determination accuracy of this LC-MS/MS method. The RE in the stability test was within 85%–115 %, and it was considered that the degradation of the compounds could be negligible under this storage condition. The RE of the diluted samples was within 85%–105% and the CV% was no more than 4%, indicating acceptable dilution integrity.

### 2.2. Free Drug Fraction of Candidates

The *f*_u_ values of CY-14S-4A83, CY-16S-4A43, and CY-16S-4A93 in rat plasma were respectively 2.98 ± 0.2%, 2.99 ± 0.2%, and 2.98 ± 0.01% when the incubation time was 30 min and were respectively 2.17 ± 0.03%, 2.99 ± 0.1%, and 2.98 ± 0.1% when the incubation time was 60 min. The *f*_u_ value of compounds with the incubation time of 30 min was comparable with that with the incubation time of 60 min, indicating that the binding of compounds to the plasma protein basically reached equilibrium within 30 min.

### 2.3. Blood-to-Plasma Concentration Ratio of Candidates

The B/P values of CY-14S-4A83, CY-16S-4A43, and CY-16S-4A93 in rat plasma were respectively 12.11 ± 0.094, 2.01 ± 0.032, and 8.08 ± 0.06 when the incubation time was 30 min and were respectively 12.34 ± 0.03, 2.53 ± 0.18, and 8.93 ± 0.02 when the incubation time was 60 min. The B/P value of compounds with the incubation time of 30 min was almost equivalent to that with the incubation time of 60 min, indicating that the distribution of the analytes into erythrocytes basically reached equilibrium within 30 min.

### 2.4. In Vitro Intrinsic Clearance of Candidates

**Figure 2:**
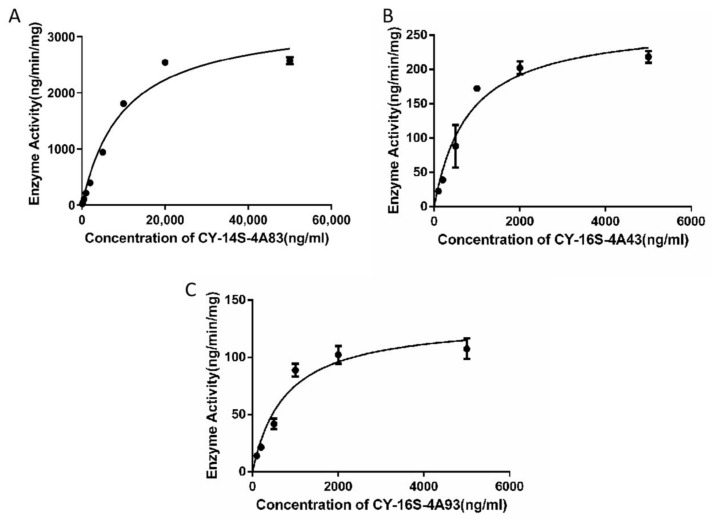
Representative Michaelis–Menten plots of the disappearance of CY-14S-4A83 (**A**), CY-16S-4A43 (**B**), and CY-16S-4A93 (**C**) via rat liver microsomes.

**Figure 3:**
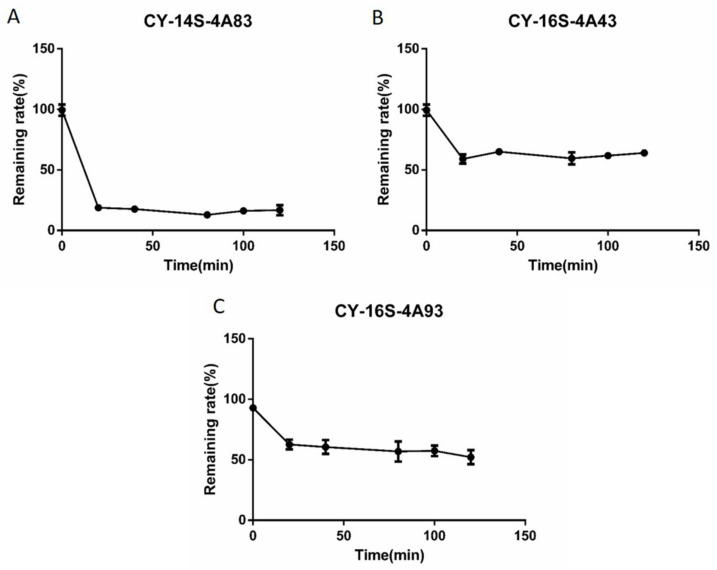
The respective remaining rates of the disappearance of CY-14S-4A83 (**A**), CY-16S-4A43 (**B**), and CY-16S-4A93 (**C**) at 0, 20, 40, 80, 100, and 120 min via rat liver microsomes.

The disappearance of CY-14S-4A83, CY-16S-4A43, and CY-16S-4A93 via rat liver microsomes followed the Michaelis–Menten kinetics in Figure 2. The in vitro hepatic intrinsic clearance parameters, such as *V*_max_, *K*_m_, and CL_int_, are listed in Table 3. The *V*_max_ of CY-14S-4A83 was highest with a *V*_max_ of 6044.0 pmol/min/mg protein, followed by CY-16S-4A43, with a *V*_max_ of 481.2 pmol/min/mg protein, and the *V*_max_ of CY-16S-4A93 was lowest, with a *V*_max_ of 220.0 pmol/min/mg protein. The *K*_m_ value of CY-14S-4A83 was highest (*K*_m_: 18.0 μM), and the *K*_m_ values of CY-16S-4A43 and CY-16S-4A93 were similar (*K*_m_: 1.4 μM vs. 1.3 μM). The CL_int_ values of CY-14S-4A83 and CY-16S-4A43 were comparable (336.0 µL/min/mg vs. 332.8 µL/min/mg), and the CL_int_ of CY-16S-4A93 was smallest, with a value of 173.8 µL/min/mg. The effects of incubation times of 0, 20, 40, 80, 100, and 120 min on the respective metabolisms of CY-14S-4A83, CY-16S-4A43, and CY-16S-4A93 are shown in Figure 3. The residual rate of each analyte was comparable within 20-120 min of incubation time, indicating that it was reasonable to set the incubation time to 20 min.

### 2.5. Pharmacokinetics of Candidates

**Figure 4:**
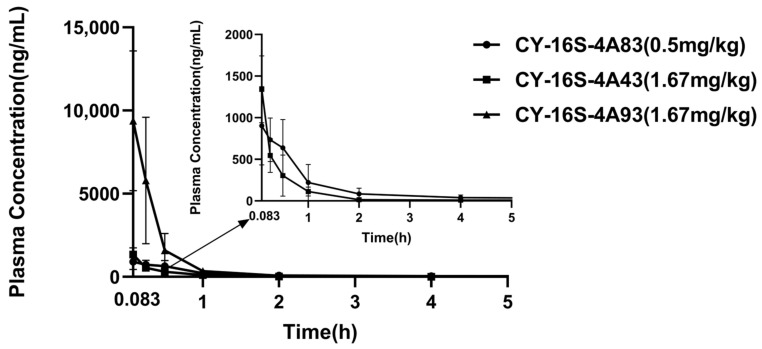
Arterial plasma concentration-time profiles of CY-14S-4A83, CY-16S-4A43, and CY-16S-4A93 after intravenous administration to rats at a single dose of 0.5 mg/kg, 1.67 mg/kg, and 1.67 mg/kg, respectively.

The validated LC-MS/MS method has been successfully employed in the PK study of CY-14S-4A83, CY-16S-4A43, and CY-16S-4A93. The concentration-time profiles are shown in Figure 4. The LC-MS/MS method can follow the PK of CY-14S-4A83 and CY-16S-4A93; i.e., the concentrations stay above the LLOQ of 10 ng/mL within 24 h. However, CY-16S-4A43 cleared faster, dropping to less than 10 ng/mL within 2–4 h. The results showed that the detection method could satisfy the determination of the concentration of these compounds for 3–5 half-life periods as the levels after that time have little significance. The relevant PK parameters are presented in Table 4. As shown in Table 4, the mean C_max_ and AUC_0–24_ of CY-14S-4A83, CY-16S-4A43, and CY-16S-4A93 were 945.9 ± 452.5 ng/mL and 1308.0 ± 461.7 ng·h/mL, 1209.6 ± 481.9 ng/mL and 668.6 ± 181.8 ng·h/mL, and 9376.2 ± 4205.6 ng/mL and 3845.1 ± 1773.4 ng·h/mL, respectively. Intravenous injection produced a rapid concentration increase, and T_max_ values of CY-14S-4A83, CY-16S-4A43, and CY-16S-4A93 were 0.2 ± 0.2 h, 0.1 ± 0.1 h, and 0.1 ± 0 h, respectively. The mean t_1/2_ values of CY-14S-4A83, CY-16S-4A43, and CY-16S-4A93 were 1.0 ± 0.2 h, 0.5 ± 0.1 h, and 0.9 ± 0.2 h, respectively. The CL and V_d_ of CY-16S-4A43 were highest (2624.6 ± 648.4 mL/h/kg and 2.03 ± 0.63 L/kg), followed by CY-16S-4A93 (500.6 ± 195.2 mL/h/kg and 0.68 ± 0.38 L/kg), and those of CY-14S-4A83 were lowest (414.5 ± 145.7 mL/h/kg and 0.56 ± 0.15 L/kg). The MRT values of CY-14S-4A83, CY-16S-4A43, and CY-16S-4A93 were 6.1 ± 1.5 h, 4.5 ± 1 h, and 2.2 ± 0.7 h, respectively.

### 2.6. PBPK Modeling of Candidates

**Figure 5:**
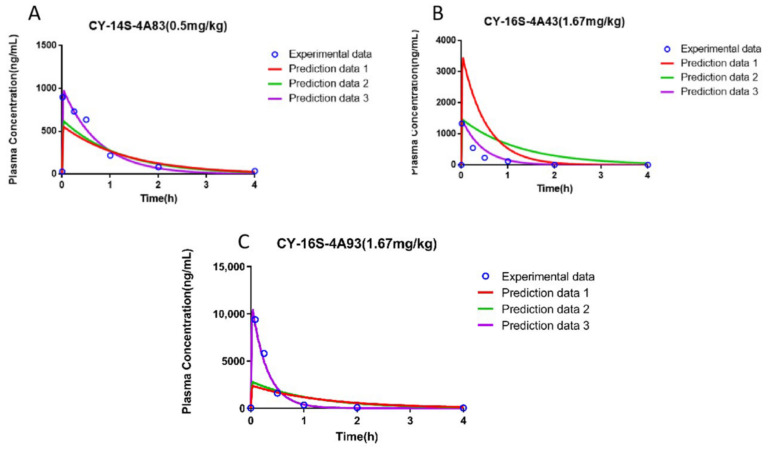
Comparison of the experimental plasma concentration-time data with the simulated plasma concentration-time profile of CY-14S-4A83 (**A**) with Kp scalar of 1 (prediction 1), Kp scalar of 0.01 (prediction 2), and Vd of 0.5 L/kg (prediction 3); CY-16S-4A43 (**B**) with Kp scalar of 1 (prediction 1), Kp scalar of 3 (prediction 2), and the added additional clearance of 5 mL/min (prediction 3); and CY-16S-4A93 (**C**) with Kp scalar of 1 (prediction 1), Kp scalar of 0.01 (prediction 2), and Vd of 0.15 L/kg (prediction 3).

The PBPK models of CY-14S-4A83, CY-16S-4A43, and CY-16S-4A93 were successfully built on the basis of the properties listed in Table 5. The log P_o:w_ and p*K*_a_ values of CY-14S-4A83, CY-16S-4A43, and CY-16S-4A93 were predicted by the ADMET Predictor, and the values were 2.497 and 5.12, 1.807 and 5.13, and 2.88 and 5.10, respectively. Figure 5 shows the simulated PK profiles of CY-14S-4A83, CY-16S-4A43, and CY-16S-4A93 after the intravenous administration to rats at a dose of 0.5 mg/kg, 1.67 mg/kg, and 1.67 mg/kg, respectively. The predicted PK parameters of CY-14S-4A83, CY-16S-4A43, and CY-16S-4A93 are listed in Table 6. Initially, the fold errors of CY-16S-4A43 and CY-16S-4A93 were both higher than the threshold value of 2, except for the fold errors of CY-14S-4A83, which were less than 2. In order to further optimize the prediction results, some parameter values have been adjusted on the basis of the experimental PK values. For the CY-16S-4A43 PBPK model, the predicted C_max_ and AUC were both two times higher than the experimental ones (prediction 1: C_max_—3437.49 ng/mL vs. 1209.6 ng/mL; AUC—1863.46 ng·h/mL vs. 668.6 ng·h/mL). The predicted C_max_ was reduced to 1456.07 ng/mL (fold error: 1.2), with the tissue–plasma partitioning coefficient scalar (Kp scalar) increasing to 3, and the AUC was reduced to 661.82 ng·h/mL (fold error: 1.01) with the adding of additional clearance of 5 mL/min. As shown in Figure 5A, although the fold errors of CY-14S-4A83 were within 2, the predicted concentration of CY-14S-4A83 was slightly lower than the experimental one (prediction 1). After optimizing, the C_max_ was increased to 620.14 ng/mL (fold error: 1.71) with the Kp scalar setting at 0.01 (prediction 2), which further increased to 972.74 ng·h/mL (fold error: 1.03) with the V_d_ setting at 0.5 L/kg (prediction 3). For the CY-16S-4A93 PBPK model (prediction 1), the predicted C_max_ was higher than the experimental one (2363.36 ng/mL vs. 9376.2 ng/mL), with a fold error of 3.97, while the predicted AUC was close to the experimental one with a fold error of 1.18. The predicted C_max_ was increased to 2754.85 ng/mL, with the Kp scalar adjusted to the minimum value of 0.01 (prediction 2), while the fold error remained larger than 2 (fold error: 3.4). In order to further optimize the model, C_max_ was increased to 10369.84 ng/mL, with a fold error of 1.11, via adjusting the V_d_ to 0.15 L/kg (prediction 3). Moreover, after the PBPK models were successfully optimized, the distributions of the candidates in the tissues were predicted, as shown in Table 7.

## 3. Discussion

In the development and optimization process of this LC-MS/MS method, we first determined and optimized the MRM parameters (Q1–Q3: 603.1–333.1, 560.1–290.0, and 553.1–187.8) according to the structure and molecular weight of the compounds. In the optimization of the chromatographic methods, the C18 chromatographic column and the elution environment of the weak acidic mobile phase were selected according to the structural characteristics (with –SO_2_ group) and the weak acidic properties of the compounds. According to the small inner diameter of the chromatographic column (2.1 × 50 mm; 1.7 μm), we chose the flow rate of 0.4 mL/min. In order to weaken the matrix effect, a smaller injection volume of 2 μL was selected. In the optimization process of the extraction method, in order to balance the lower limit of quantitation, the matrix effect, and the extraction recovery, the 1:4 organic solvent protein precipitation method for pretreatment was finally chosen.

It is well known that preclinical PK, pharmacology, and toxicology research forms a preclinical new drug-screening and evaluation system to evaluate the risk–benefit ratio of candidate drugs before the first-in-human clinical study. Moreover, activity and toxicity are closely related to the concentration of compounds in the body. In preliminary pharmacologic studies, the furoxan/coumarin hybrids displayed potent anticancer activities with the IC_50_ (half maximal inhibitory concentration) of 0.5–143 nM and low toxicities [24]. In order to understand the PK behavior of the compounds in vivo, the ADME properties were explored using the LC-MS/MS method and PBPK modeling. Owing to the free drug principle that only a free drug molecule can pass through the cell membrane to exert effects, *f*_u_ is an important factor affecting the drug behaviors of ADME [41]. The *f*_u_ values of CY-16S-4A43, CY-16S-4A93, and CY-14S-4A83 were very low (2.99 ± 0.1%, 2.98 ± 0.1%, and 2.17 ± 0.03%, respectively). It is indicated that we need to pay more attention to the dosage setting in the follow-up research to avoid possible adverse reactions, because small *f*_u_ changes in compounds with low *f*_u_ may lead to a sharp increase in the free compound in vivo.

B/P is a parameter that measures the distribution of compounds to the cells in the blood. The blood clearance rate can be calculated on the basis of B/P and the plasma clearance rate. The B/P values of CY-14S-4A83, CY-16S-4A43, and CY-16S-4A93 were all greater than 1, indicating that the binding of compounds to plasma proteins was less than that to blood cells, and the plasma clearance rate would overestimate the blood clearance rate. The metabolic liver plasma clearance rates of CY-14S-4A83, CY-16S-4A43, and CY-16S-4A93 in rats were 2.75 mL/min, 3.73 mL/min, and 2.13 mL/min, respectively, and the values of liver blood clearance were calculated to be 0.23 mL/min, 1.87 mL/min, and 0.24 mL/min, respectively.

The PK properties of CY-14S-4A83, CY-16S-4A43, and CY-16S-4A93 in rats were explored and would intuitively reflect the PK process of each drug in the body. The AUC_0–∞_ and the AUC_0–24_ were comparable, reflecting that the compounds were completely eliminated from the body within 24 h. The t_1/2_ of CY-16S-4A43 was the shortest and its CL was the highest, which was in accordance with the highest CL_int_ of CY-16S-4A43 compared with that of the other two compounds. Moreover, the concentrations of the candidates in vivo were higher than the range of IC_50_ within 1 h, and the plasma concentration dropped to less than 50 ng/mL within 4 h (Figure 4), indicating a feasible dosage setting.

In order to further explore the influence of the ADME characteristics and physicochemistry properties on the disposal of the compounds in vivo, the PBPK models of CY-14S-4A83, CY-16S-4A43, and CY-16S-4A93 were constructed with in vitro parameters. As shown in Table 6, the predicted PK parameters of the CY-14S-4A83 PBPK model (prediction 1) were within the range of one-half of to two times the experimental value. Additionally, CL was further predicted (2.75 mL/min), which was within two times the experimental value (1.73 mL/min). However, the predicted PK parameters of the CY-16S-4A43 PBPK model (prediction 1) were overestimated compared with the experimental ones, while the predicted CL (3.73 mL/min vs. 10.94 mL/min) and V_d_ (0.47 L/kg vs. 2.03 L/kg) were underestimated, indicating that there may be tissue distribution and additional elimination pathways in the PK process of CY-16S-4A43 in vivo. After increasing Kp (1 to 3) and adding an additional CL of 5 mL/min in PBPK modeling (prediction 3), the predicted PK parameters were decreased, with fold errors reduced to 1.17 and 1.01, and the predicted CL and V_d_ were increased to 10.5 mL/min and 1.14 L/kg, respectively (fold error: 1.04 and 1.78), which supported our speculation that there may be tissue accumulation and other elimination pathways. Moreover, the predicted Kp in various tissues were more than 1 (Table 7), demonstrating the tissue distribution of CY-16S-4A43. For the CY-16S-4A93 PBPK model (prediction 1), the predicted C_max_ was underestimated by about 74.8% relative to the experimental C_max_, while the fold error of the predicted AUC was within 2, and the CL was equivalent to the experimental value (2.13 mL/min vs. 2.08 mL/min). We speculated that the underestimated C_max_ may be related to the efflux transporter, resulting in less distribution in tissues. The fold error of the predicted C_max_ was within 2, with the V_d_ reduced to 0.15 L/kg (prediction 3), which was much less than the experimental value (0.68 L/kg). While the predicted V_d_ of 0.70 L/kg (prediction 1) was similar to the experimental value of 0.68 L/kg, it was thought that this may be related to the large individual differences in the experimental values (C_max_: *p* = 0.0028; V_d_: *p* = 0.0074). The individual variation in C_max_ was more than five times.

Solubility is an important factor affecting the absorption and distribution of compounds in the body. In previous dissolution experiments, the three compounds were soluble only in dimethyl sulfoxide (DMSO). Given that the amount of DMSO in intravenous preparations cannot be higher than 10%, dosages of 0.5 mg/kg and 1.67 mg/kg were applied. Moreover, the PK experiments of compounds in rats via oral administration were also conducted, and the results showed that the absorption was poor and that the plasma drug concentration was below the detection limit. In order to improve the low solubility of the candidates, it may be necessary to take further measures, such as structural modifications, the selection of better cosolvents or emulsifiers, or using dosage forms such as microspheres, microcapsules, or clathrates in the follow-up preclinical studies.

## 4. Materials and Methods

### 4.1. Chemicals and Reagents

**Figure 6:**
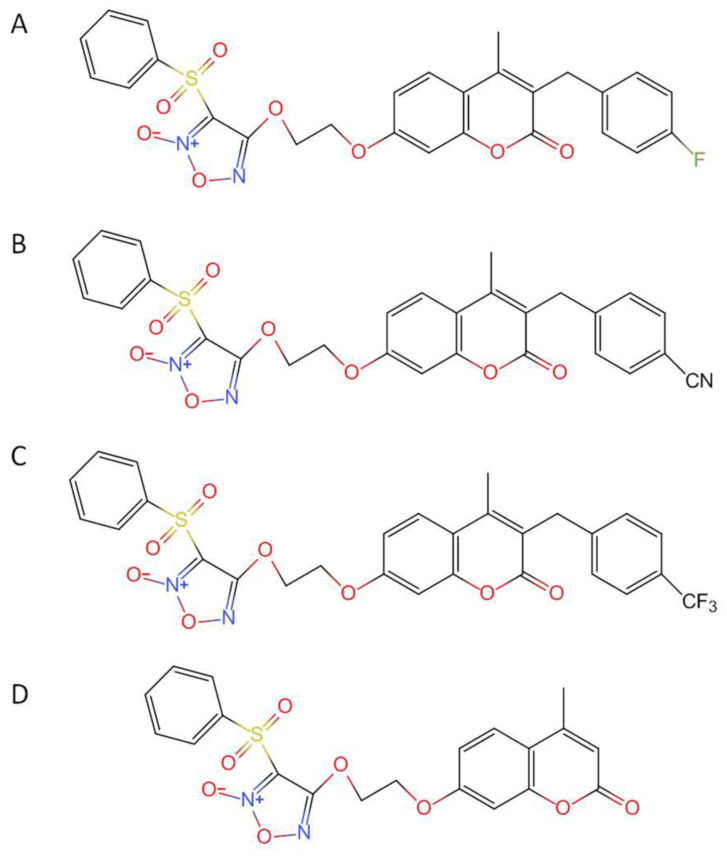
Structures of CY-14S-4A83 (**A**), CY-16S-4A43 (**B**), CY-16S-4A93 (**C**), and IS (**D**).

Three furoxan/coumarin hybrids, CY-14S-4A83, CY-16S-4A43, and CY-16S-4A93, were synthesized, as shown in Figure 6. CY-11S-1A26 was used as an internal standard (IS). Pooled IGS Sprague Dawley rat liver microsomes (male at 20 mg/mL) were purchased from XENTECH (Paris, France). Coenzyme II-reduced tetrasodium salt (β-NADPH) was obtained from Roche (Mannheim, Germany). Distilled and deionized water was purified from the Milli-Q water system (Millipore, Molsheim, France). Acetonitrile and methanol were HPLC grade and acquired from Merck KGaA (Darmstadt, Germany). Other reagents were of analytical grade.

### 4.2. Animals

The animal experimental protocol was reviewed and approved by the Animal Ethics Committee of College of Pharmacy of Fudan University. Specifically, 18 healthy male Sprague Dawley (SD) rats, weighing 280–310 g, were provided and raised by the Animal Experiment Center of the College of Pharmacy of Fudan University. The animal room was maintained at 22 ± 1 °C and kept in a 12 h normal light/dark cycle. The laboratory food and tap water were available ad libitum for rats.

### 4.3. Free Drug Fraction Determinations

The *f*_u_ of furoxan/coumarin hybrids in plasma was measured through the ultrafiltration method by using Amicon Ultra-15 ultrafiltration centrifuge tube (0.5 mL, 3-kDa membrane, Millipore, Bedford, MA, USA). Two batches of CY-14S-4A83, CY-16S-4A43, and CY-16S-4A93 solution were prepared with the fresh rat plasma to obtain final concentrations of 1 μg/mL, 1 μg/mL, and 5 μg/mL, respectively. One batch was incubated in a shaking water bath at 37 °C for 30 min, and the other batch was incubated for 60 min. After incubation, a plasma sample was transferred into ultrafiltration tube and centrifuged at 10,000 rpm for 20 min. The free compound in the filtrated fraction was determined directly by LC-MS/MS. All experiments were performed in triplicate.

### 4.4. Blood-to-Plasma Concentration Ratio Determinations

CY-14S-4A83, CY-16S-4A43, and CY-16S-4A93 were spiked in fresh heparinized rat blood to yield final concentrations of 1 μg/mL, 1 μg/mL, and 5 μg/mL, respectively. Two batches of blood samples were prepared in parallel. One batch of the blood samples was then incubated in a shaking water bath at 37 °C for 30 min, and the other batch was incubated for 60 min. After incubation, one 150 μL aliquot of blood samples was centrifuged at 1500 rpm for 5 min, and the concentrations of the testing compound in the resulting plasma portion after extraction were determined by LC-MS/MS. In parallel, another 50 uL aliquot of the blood incubation sample was transferred into an Eppendorf tube and lysed by three-cycle freeze/thaw method. After the cell-lysis and extraction, the concentration of testing compound in the whole blood sample was determined by LC-MS/MS. The B/P was determined directly by the ratio of drug concentration in the whole blood to the plasma concentration. All experiments were performed in triplicate.

### 4.5. In Vitro Intrinsic Clearance (CL_int_) Determinations

The CL_int_ of CY-14S-4A83, CY-16S-4A43, and CY-16S-4A93 in pooled hepatic microsomes was determined at concentration ranges of 0.1–50 μg/mL, 0.1–5 μg/mL, and 0.1–5 μg/mL, respectively. The incubation mixture with a final volume of 50 μL contained the testing compound, rat liver microsomes (0.2 mg protein/mL), phosphate buffer (0.1 mM, pH 7.4), and NADPH (1 mM). Before adding NADPH, the reaction mixture was preincubated in a shaking water bath at 37 °C for 5 min. 200 μL of methanol–acetonitrile mixture (1:1) containing IS was added to terminate the reaction after incubation for 20 min. Concentration of the testing compound was determined by LC-MS/MS after centrifugation at 14,000 rpm for 15 min. Meanwhile, the effects of incubation times of 0, 20, 40, 80, 100, and 120 min on CY-14S-4A83, CY-16S-4A43, and CY-16S-4A93 metabolisms were examined at a final concentration of 0.1 μg/mL. All experiments were performed in triplicate.

### 4.6. Pharmacokinetic Study

Rats were randomly divided into three groups, namely A, B, and C, with six rats in each group. CY-14S-4A83 was intravenously administrated to rats in group A at a single dose of 0.5 mg/kg, and CY-16S-4A43 and CY-16S-4A93 were intravenously administrated to rats in groups B and C at a single dose of 1.67 mg/kg each. Blood samples (0.15–0.2 mL) were collected through the femoral artery into an Eppendorf tube moistened with heparinized 0.9% NaCl before intravenous injection (0 h) and at 0.083, 0.25, 0.5, 1, 2, 4, 8, 12, and 24 h after intravenous injection. The blood sample was immediately centrifuged at 3000 rpm for 10 min at a temperature of 4 °C, and the upper plasma sample was collected and stored at −80 °C until analysis.

The plasma samples were thawed at room temperature before determination. Further, 20 μL of plasma was pipetted into a 1.5 mL Eppendorf tube, and 80 μL of methanol–acetonitrile (1:1, *v*/*v*) solution containing 200 ng/mL IS was added to precipitate the proteins. The contents were mixed thoroughly via vortexing the plasma sample for 40 s. The supernatant was aspirated for LC-MS/MS analysis after centrifugation at 14,000 rpm for 15 min.

### 4.7. LC-MS/MS Analysis Method

The LC-MS/MS analysis was performed on an Agilent 1260 UPLC (Agilent, Santa Clara, CA, USA), coupled to an Agilent 6490 Triple Quad mass spectrometer (Agilent, USA) with an electrospray ionization (ESI) source. An Agilent ZORBAX Extend C18 column (2.1 × 50 mm; 1.7 μm) was used for separation, with a flow rate of 0.4 mL/min, and the column temperature was set to 35 °C. The mobile phases were composed of (A) 0.1% formic acid in 100% water (pH = 2.79) and (B) 0.1% formic acid in 100% acetonitrile. The gradient elution was performed as below: 50% B was kept for 1.0 min, followed by a linear increasing to 95% B during 2.0 min and maintained at 95% B for 5.0 min, then decreased to 50% B in 0.1 min and maintained for 2.9 min. The injection volume was set to 2 μL.

The mass parameters were optimized and shown as follows: capillary voltage at 4000 V, nozzle voltage at 500 V, gas temperature at 250 °C, gas flow at 12 L/min, nebulizer at 35 psi, sheath gas temperature at 300 °C, and sheath gas flow at 11 L/min. Multiple reaction monitoring (MRM) was used to monitor CY-14S-4A93, CY-16S-4A43, CY-14S-4A83, and CY-11S-1A26 (IS) in the positive ion mode. The detailed MRM transitions and collision energy are listed in Table 8. Data acquisition and processing were performed on Agilent MassHunter Workstation software (Agilent, USA).

The concentration of the testing compound was calculated by using the calibration curves with seven dots (10, 25, 50, 100, 250, 500, and 1000 ng/mL). The calibration line was constructed by plotting the peak area ratio (*y*-axis) of analytes to IS versus the nominal concentration (*x*-axis). The specificity and selectivity were investigated by comparing the chromatograms of blank plasma samples with the corresponding samples spiked with testing compounds. The signal-to-noise ratio (S/N) of the lower limit of quantification (LLOQ) was at least at 10. The linearity of the calibration graph was validated, and the calculated concentrations of the calibration standards should be all within ±15% of the nominal value, except for the LLOQ of ±20%. The linear correlation coefficient (R^2^) should be 0.9900 or greater. The intra- and interday precisions and accuracy of the method were assessed by determining the quality control (QC) samples of 8, 4, and 0.2 μg/mL (six samples at each concentration level) over 3 consecutive days. The coefficient of variation (CV) should not exceed 20% at LLOQ and should be within 15% for QCs. The relative error (RE) should be within 80%–120% at LLOQ and within 85%–115% for QCs. Recovery was assessed by comparing peak area of nominal QC plasma samples with peak area of the spike-after extraction samples (six samples at each concentration level). The matrix effect was evaluated by comparing peak area of the spike-after extraction samples with that of the analytes spiked in pure water (six samples at each concentration level). The recovery and matrix factor should be within 85%–115%. The variation in recovery and matrix effect should be less than 15%. The dilution integrity test was performed for samples exceeding the upper limit of quantification (ULOQ). QCs were prepared (5 samples at each concentration level) and frozen at 80 °C for one week. LC-MS/MS analysis was performed to calculate the stability (RE). Samples of 25 μg/mL (*n* = 6) were diluted with blank matrix to 0.5 μg/mL, which was within the calibration line range, and the RE should be within 85–115%.

### 4.8. PBPK Modeling

PBPK models were constructed with physical chemistry and in vitro PK parameters, such as molecular weight (MW), octanol–buffer partition coefficient (log P_o:w_), compound type, p*K*_a_, B/P, *f*_u_, and CL_int_, using Simcyp (Simcyp Rat Version 16, Sheffield, UK). Two physical chemistry parameters of log P_o:w_ and p*K*_a_ were predicted on the basis of the structure of the chemicals by using the ADMET Predictor (v.9.0, Lancaster, http://www.simulations-plus.com/, accessed on 9 September 2019). Other parameters, including B/P, *f*_u_, and CL_int_, were obtained through in vitro experiments as mentioned above. The time course of xenobiotic concentration in plasma was simulated, and the PBPK models were further optimized on the basis of the experimental in vivo PK parameters (AUC, C_max_, and time to reach C_max_ (T_max_)). The prediction accuracy of the PBPK model was evaluated by introducing the deviation (fold error) of the predicted PK parameters and comparing them with the experimental ones. The equation of the fold error is shown below. A simulation with fold errors no greater than 2 is acceptable [36,42].

(1) $$\mathrm{fold}\;\mathrm{error}=\frac{\mathrm{observed}\;\mathrm{parameter}}{\mathrm{predicted}\;\mathrm{parameter}};\mathrm{if}\;\mathrm{observed}\;\mathrm{value}>\mathrm{predicted}\;\mathrm{value}$$

(2) $$\mathrm{fold}\;\mathrm{error}=\frac{\mathrm{predicted}\;\mathrm{parameter}}{\mathrm{observed}\;\mathrm{parameter}};\mathrm{if}\;\mathrm{predicted}\;\mathrm{value}>\mathrm{observed}\;\mathrm{value}$$

### 4.9. Data Analysis

The CL_int_ for the disappearance of CY-14S-4A83, CY-16S-4A43, and CY-16S-4A93 was calculated by dividing the *V*_max_ (maximum reaction velocity) by the *K*_m_ (concentration of substrate with the reaction velocity to 50% *V*_max_). *V*_max_ and *K*_m_ were estimated via the nonlinear regression of the Michaelis–Menten model by using GraphPad Prism (Version 6.01, http://www.graphpad.com/). The PK parameters were determined from drug plasma concentration-time data by employing a noncompartmental approach using Phoenix WinNonlin (version 3.0). T_max_, C_max_, AUC, area under the plasma concentration-time curve during the period from 0 to infinity (AUC_0–∞_), area under the plasma concentration-time curve from 0 to 24 h (AUC_0–24_), elimination half-life (t_½_), mean residence time (MRT), clearance (CL), and the steady state volume of distribution (V_d_) were estimated.

## 5. Conclusions

A LC-MS/MS method was developed and successfully applied in the preclinical study of the novel furoxan/coumarin hybrids: CY-14S-4A83, CY-16S-4A43, and CY-16S-4A93. The method was validated and possessed high sensitivity, wide linearity, good specificity, and no interferences from endogenous substances. The PK properties of the three furoxan/coumarin hybrids were analyzed through the in vitro ADME data and in vivo PK parameters. Detailed information from the PBPK models was explored and indicated that tissue distribution and other elimination pathways may be concerned in the PK process of CY-16S-4A43. Structural modifications or dosage form adjustment may be needed in the follow-up preclinical research to improve the solubility and bioavailability of the candidate compounds. In summary, the preclinical PK study of the novel furoxan/coumarin hybrids using the LC-MS/MS method and PBPK modeling may inspire new ideas in the three Rs practice in preclinical PK studies.

## Figures and Tables

**Table 1:** Calibration curve parameters of CY-14S-4A83, CY-16S-4A43, and CY-16S-4A93.

Calibration Curve Parameters	CY-14S-4A83	CY-16S-4A43	CY-14S-4A93
Linearity equation	y = 0.038713x − 0.000446	y = 0.045595x + 0.000448	y = 0.028248x − 0.000252
R^2^	0.99994	0.99986	0.99362
LLOQ (ng/mL)	10	10	10

**Table 2:** Intra- and interday precisions and the accuracy, recovery, and matrix effects of CY-14S-4A83, CY-16S-4A43, and CY-16S-4A93.

Property	Concentration (ng/mL)	CY-14S-4A83	CY-16S-4A43	CY-14S-4A93
Intraday Precision (%) (*n* = 6)	10–800	3.4–8.5	2.3–5	1.6–7.8
Interday Precision (%) (*n* = 6)	10–800	2.9–7.9	3.6–8.8	1.2–12.5
Intraday Accuracy (%) (*n* = 6) ^a^	10–800	102.9–108.2	106.2–108.3	98.8–109
Interday Accuracy (%) (*n* = 18) ^a^	10–800	101.7–107.8	105.4–108.7	103.4–106.1
Recovery (%) (*n* = 6)	20–800	99.9–114.4	85.2–87.5	101.8–112.5
CV (%)		8.8	1.4	5
Matrix effect (%) (*n* = 6)	20–800	86.4–103.7	86.3–94.5	88.4–113.9
CV (%)		10.3	5.2	12.6
Stability (%) (*n* = 5)	20–800	88–94.4	86–99.5	86.5–94.7
CV (%)		3.8	7.3	4.7
Dilution integrity (*n* = 6) ^a^	500	89.2 ± 3.2	102.3 ± 3.5	94.4 ± 2.7

^a^ Data presented as mean ± standard deviation.

**Table 3:** Kinetic constants for the disappearance of CY-14S-4A83, CY-16S-4A43, and CY-16S-4A93 in rat hepatic microsomes.

Compound	V_max_ (pmol/min/mg Protein) ^a^	*K*_m_ (μM) ^a^	CL_int_ (µL/min/mg)
CY-14S-4A83	6044.0 ± 290.8	18.0 ± 2.3	336.0
CY-16S-4A43	481.2 ± 30.0	1.4 ± 0.3	332.8
CY-14S-4A93	220.0 ± 14.1	1.3 ± 0.2	173.8

^a^ Data presented as mean ± standard deviation.

**Table 4:** PK parameters of CY-14S-4A83, CY-16S-4A43, and CY-16S-4A93 after intravenous administration to rats.

Parameters	Unit	CY-14S-4A83	CY-16S-4A43	CY-16S-4A93
		0.5 mg/kg	1.67 mg/kg	1.67 mg/kg
AUC_(0–∞)_	ng·h/mL	1343.6 ± 475.4	672.6 ± 181.6	3854.6 ± 1774.3
AUC_(0–24 h)_	ng·h/mL	1308.0 ± 461.7	668.6 ± 181.8	3845.1 ± 1773.4
C_max_	ng/mL	945.9 ± 452.5	1209.6 ± 481.9	9376.2 ± 4205.6
T_max_	h	0.2 ± 0.2	0.1 ± 0.1	0.1 ± 0
V_d_	L/kg	0.56 ± 0.15	2.03 ± 0.63	0.68 ± 0.38
CL	mL/h/kg	414.5 ± 145.7	2624.6 ± 648.4	500.6 ± 195.2
t_1/2_	h	1.0 ± 0.2	0.5 ± 0.1	0.9 ± 0.2
MRT	h	6.1 ± 1.5	4.5 ± 1	2.2 ± 0.7

Data presented as mean ± standard deviation.

**Table 5:** Parameters of CY-14S-4A83, CY-16S-4A43, and CY-16S-4A93 used in the PBPK modeling.

Parameters	CY-14S-4A83	CY-16S-4A43	CY-14S-4A93
MW (g/mol)	552.53	559.55	602.54
log P_o:w_	2.497	1.807	2.88
compound type	Monoprotic Acid	Monoprotic Acid	Monoprotic Acid
p*K*_a_	5.12	5.13	5.1
B/P	12	2	9
*f*_u_	0.02	0.03	0.03
LM CL_int_ (µL/min/mg)	336.033	332.8	173.83

**Table 6:** Prediction parameters of the PBPK models of CY-14S-4A83, CY-16S-4A43, and CY-16S-4A93.

Compounds	Parameters	C_max_ (ng/mL)	AUC (ng·h/mL)
CY-14S-4A83	Experimental data	945.9 ± 452.5	1308.0 ± 461.7
	Prediction data 1 ^a^	553.83	757.87
	Fold Error	1.71	1.73
	Prediction data 2 ^b^	620.14	757.86
	Fold Error	1.53	1.73
	Prediction data 2 ^c^	972.74	757.87
	Fold Error	1.03	1.73
CY-16S-4A43	Experimental data	1209.6 ± 481.9	668.6 ± 181.8
	Prediction data 1 ^a^	3437.49	1863.46
	Fold Error	2.84	2.79
	Prediction data 2 ^d^	1456.07	1863.45
	Fold Error	1.2	2.79
	Prediction data 3 ^e^	1410.01	661.82
	Fold Error	1.17	1.01
CY-16S-4A93	Experimental data	9376.2 ± 4205.6	3845.1 ± 1773.4
	Prediction data 1 ^a^	2363.36	3263.5
	Fold Error	3.97	1.18
	Prediction data 2 ^f^	2754.85	3263.47
	Fold Error	3.4	1.18
	Prediction data 2 ^g^	10,369.84	3263.57
	Fold Error	1.11	1.18

^a^ Parameters inputted into the PBPK modeling as listed in Table 5; ^b^ PBPK model with the Kp scalar of 0.01; ^c^ PBPK model with the Vd of 0.5 L/kg; ^d^ Kp scalar was changed to 3; ^e^ Kp scalar was changed to 3, and additional clearance of 5 mL/min was added in PBPK modeling; ^f^ PBPK model with the Kp scalar of 0.01; ^g^ Vss was adjusted 0.15 L/kg.

**Table 7:** Predicted distribution of CY-14S-4A83, CY-16S-4A43, and CY-16S-4A93 in tissues of rats.

Tissues		Kp	
	CY-14S-4A83	CY-16S-4A43	CY-16S-4A93
Adipose	0.00	0.02	0.00
Bone	0.00	0.83	0.00
Brain	0.00	1.51	0.00
Gut	0.00	1.49	0.00
Heart	0.00	1.37	0.00
Kidney	0.00	1.35	0.00
Liver	1.00	1.32	1.00
Lung	0.00	1.43	0.00
Muscle	0.00	1.29	0.00
Skin	0.00	1.47	0.00
Spleen	0.00	1.35	0.00

**Table 8:** Optimal MRM parameters for CY-14S-4A93, CY-16S-4A43, CY-14S-4A83, and CY-11S-1A26.

Compound	Q1/Da	Q3/Da	DT/Msec	Frag/V	CE/V
CY-14S-4A93	603.1	333.1	75	380	33
CY-16S-4A43	560.1	290.0	75	380	29
CY-14S-4A83	553.1	187.8	75	380	37
CY-11S-1A26(IS)	445.1	175.9	75	380	25

## Data Availability

No new data were created or analyzed in this study. Data sharing is not applicable to this article.

## Sample Availability

Samples of the compounds of CY-14S-4A83, CY-16S-4A43 and CY-16S-4A93 are available from the authors.
